# Curcumin/Liposome Nanotechnology as Delivery Platform for Anti-inflammatory Activities via NFkB/ERK/pERK Pathway in Human Dental Pulp Treated With 2-HydroxyEthyl MethAcrylate (HEMA)

**DOI:** 10.3389/fphys.2019.00633

**Published:** 2019-06-11

**Authors:** Bruna Sinjari, Jacopo Pizzicannella, Marco D’Aurora, Romina Zappacosta, Valentina Gatta, Antonella Fontana, Oriana Trubiani, Francesca Diomede

**Affiliations:** ^1^Department of Medical Oral and Biotechnological Sciences, University “G. d’Annunzio”, Chieti, Italy; ^2^ASL02 Lanciano-Vasto-Chieti, “Ss. Annunziata” Hospital, Chieti, Italy; ^3^Department of Psychological, Health and Territorial Sciences, University “G. d’Annunzio”, Chieti, Italy; ^4^Department of Pharmacy, University “G. d’Annunzio”, Chieti, Italy

**Keywords:** liposome curcumin, human dental pulp stem cells, inflammation, methacrylate, NFkB/ERK/pERK

## Abstract

Curcumin, primary component of the spice turmeric extracted from the rhizomes of *Curcuma longa*, represents the major anti-oxidant and anti-inflammatory substance found in turmeric, acting thought various mechanisms not completely understood. Curcumin modulates cytokines, growth factors, transcription factors, inflammatory molecules and cell signaling pathways. During restorative dentistry practice, free resin monomers of 2-hydroxyethyl methacrylate (HEMA) propagate through dentin micro-channel and pulp into the bloodstream affecting cellular integrity. The study highlights the significance of application of curcumin bioactive component into liposomal formulations (CurLIP) to restore the homeostasis of dental pulp stem cells (hDPSCs) in response to 3 and 5 mmol L^–1^ HEMA treatment. Cell proliferation in combination with changes of the morphological features, proinflammatory cytokines secretion as Interleukin (IL) 6, IL8, Monocyte Chemoattractant Protein-1 (MCP1) and Interferon-gamma (IFNγ) were assayed along with the nuclear factor (NF)-kB, an inducible transcription factor involved in the activation of several cell processes associated to extracellular signal-regulated kinases (ERK) and posphorylated (p-) ERK pathway. Our results showed a decreased cell proliferation, morphological changes and upregulation of IL6, IL8, MCP1 and IFNγ in presence of 3 and 5 mmol L^–1^ HEMA treatment. CurLIP therapy in hDPSCs provokes an increase in cell proliferation and the block of inflammatory cytokines secretion through the inhibitory regulation of NFkB/ERK and pERK signaling cascade. The natural nanocarrier CurLIP influences numerous biochemical and molecular cascades causing anti-inflammatory properties in response to HEMA treatment in human dental pulp stem cells, representing an innovative endodontic formulation able to improve the quality of dental care with a major human community impact.

## Introduction

In the world, the 36% of the population have dental caries/decay in their permanent teeth and the 9% of the population in their baby teeth (Vos et al., 2012).

To restore teeth damage are predominantly used dental composites that contain viscous methacrylate monomers such as 2,2-bis[4-(2-hydroxy-3-methacrylyloxy-propoxy)phenyl] propane (bis-GMA) and urethane dimethacrylate (UDMA), in addition to hydrophilic monomers such as 2-hydroxyethyl methacrylate (HEMA) and triethylene glycol dimethacrylate (TEGDMA; Trubiani et al., 2012b; Diomede et al., 2014). Resin monomers are released during the incomplete polymerization of composite resins and can penetrate into the pulp tissue exerting cytotoxic effects and inducing the release of proinflammatory cytokines. HEMA and TEGDMA impede odontogenic differentiation and the mineralization of apical papilla stem cells as well as the inflammatory cytokines secretion (Trubiani et al., 2010; Bakopoulou et al., 2012).

In this scenario, many interdisciplinary studies have been addressed in order to favor the repair of cells and tissues damages. In particular, in endodontic treatment, several natural compounds and herbal products are acquiring a great popularity for their properties (Ravishankar et al., 2017). Curcuma longa is a plant belonging to the family of Zingiberaceae whose rhizome, source of turmeric, has been used in cooking, cosmetics and medical treatments (Vaughn et al., 2016) from considerable time. The curcuminoids present in the rhizome are a mixture of curcumin, demethoxycurcumin, and bisdemethoxycurcumin (Gangemi et al., 2015).

Curcumin possesses, *via* the activation of various cellular pathways (Mazidi et al., 2016), beneficial properties including anti-inflammatory, antioxidant, antineoplastic, pro- and antiapoptotic, antiangiogenic, immunomodulatory and antimicrobial effects. However, uncontrolled inflammation is the cause of a wide range of pathological conditions, including cardiovascular diseases, psoriasis, multiple sclerosis, rheumatoid arthritis and inflammatory bowel disease (Soltani et al., 2019). The curcumin treatment is able to inhibit the proinflammatory transcription factors, including NFkB, and to activate peroxisome proliferator activated receptor-gamma (PPARγ) cell signaling pathway (Um et al., 2013) and to diminish NFkB-dependent production of proinflammatory cytokines, such as tumor necrosis factor-α, IL6 and Macrophage Inflammatory Protein 2 (MIP2; Zingg et al., 2013).

For its chemical structure, curcumin is soluble in methanol, ethanol, dimethyl sulfoxide (DMSO), and acetone and much less in water (Ghosh et al., 2015); it is rapidly metabolized, with poor photo stability, which limit its use as an effective therapeutic agent (Chang et al., 2018; Panahi et al., 2018). Authors reported that these limitations can be overcome through liposomes that, solubilizing the curcumin in the phospholipidic bilayer, enable the delivery of curcumin in an aqueous medium and significantly improve the curcumin effect (Chang et al., 2018).

Nanotechnology represents a great promise in modern science and medicine. A nanoparticle (NP), having size ranging from 1 to few hundred of nanometers, is an example of nanotechnology. Recently, NPs have been considered as novel candidates to improve the intracellular drug delivery, to reach subcellular targeting and cross inaccessible anatomical and physiological barriers (Dende et al., 2017). Dutta and Ikiki (2013) reviewed different drug delivery strategies for curcumin such as nanosuspension, nanoemulsion, solid-lipid NPs and hydrogel NPs.

Human dental pulp, a soft connective tissue containing the pulp tissue of the tooth, is considered an interesting source of adult stem cells, due to the low-invasive isolation procedures (Paino et al., 2017), high content of stem cells and peculiar embryological origin. Deriving from neural crest, human dental pulp stem cells (hDPSCs) might represent an interesting cellular model to study response to different molecules (Pisciotta et al., 2018).

Starting from these preconditions, Curcumin loaded liposome (CurLIP) treatment might offer the opportunity to ameliorate, during vital pulp therapy, the acute phase of inflammation process restoring pulp tissue homeostasis. The aim of the present study is to evaluate the protective effect of the natural nanocarrier CurLIP in response to HEMA treatment in human dental pulp stem cells.

## Materials and Methods

### Curcumin Loaded Liposomes

Liposomes were prepared according to the following protocol. An appropriate aliquot of POPC (1-palmitoyl-2-oleoyl-phosphatidylcholine, Avanti Polar Lipids, Alabama, United States), dissolved in chloroform, was put in a round-bottomed flask and dried in a rotary evaporator under reduced pressure at 40°C to form a thin lipid film on the inside wall of the flask. The phospholipid film was kept at 4°C overnight before rehydration with PBS buffer (pH 7.4) and sonication for 30 min (Viale et al., 2016; Mammana et al., 2019). Liposomes were sterilized under UV lamp for 2 h. Then an appropriate amount of curcumin in DMSO was added to the resulting liposomal suspension in order to obtain a POPC to curcumin molar ratio of 25:1. For the *in vitro* test POPC and curcumin concentrations in the liposomal suspension were 10 mmol L^–1^ and 0.4 mmol L^–1^, respectively (molar ratio of 25:1). 100 μL of this liposomal suspension (with and without curcumin) were added to a final volume of medium of 2 mL in order to obtain a curcumin concentration of 0.02 mmol L^–1^. For ζ-potential and dimensional analysis POPC and curcumin concentrations in the liposomal suspension were 0.25 mmol L^–1^ and 0.01 mmol L^–1^, respectively (molar ratio of 25:1).

### Determination of Curcumin-Loading Into POPC Liposome

Liposomes were prepared according to the above-mentioned protocol (Viale et al., 2016). In order to evaluate the percentage of embedded curcumin and therefore remove the curcumin eventually unentrapped into liposomes, a filtration of the liposomal suspension through a Sephadex G-25 column was performed. Sephadex columns were eluted with H_2_O milliQ and curcumin entrapped into columns was quantified by UV-Vis spectrophotometry (e_434*nm*_ = 10,997).

### Cell Culture Establishment

hDPSCs were collected from the dental pulp of non-carious third molars extracted for orthodontic purpose in good health patients, as previously described (Diomede et al., 2017a). In the present study three patients have been enrolled to isolate hDPSCs. hDPSCs spontaneously migrated from tissue and were cultured using MSCGM-CD medium (mesenchymal stem cell growth medium chemically defined; Lonza, Basel, Switzerland) maintaining in an incubator at 37°C in a humidified atmosphere of 5% CO2 in air. All experiments were performed in triplicate. Each cell population (passage 2) has been used for all following experiments performed in triplicate. Cells were treated with HEMA 3 and HEMA 5 mmol L^–1^ for 48h and pretreated with CurLIP for 24h. The experimental groups were divided as following reported:

– hDPSCs without treatment (used as control – CTRL);
– hDPSCs treated with HEMA 3 mmol L^–1^;
– hDPSCs treated with HEMA 5 mmol L^–1^;
– hDPSCs treated with Curcumin loaded liposomes (CurLIP);
– hDPSCs treated with CurLIP and HEMA 3 mmol L^–1^;
– hDPSCs treated with CurLIP and HEMA 5 mmol L^–1^.

### MTT Assay

The effects on the viability of hDPSCs treated with CurLIP, HEMA 3 and HEMA 5 mmol L^–1^ were evaluated by means of the 3-(4,5-dimethylthiazolyl-2)-2,5-diphenyltetrazoliumbromide (MTT) method. 2 × 10^3^ cells/well were placed in a 96-well tissue culture plates and incubated at 37°C for 24, 48 and 72 h. At each time point, MTT solution (20 μl; Promega, Milan, Italy) was added to each well to detect the metabolic activity of the cells. All plates were cultured in the dark for 3 h at 37°C (Diomede et al., 2016a). Supernatants were read at 650 nm wavelength using a microplate reader (Synergy HT, BioTek Instruments, Winooski, VT, United States; Cavalcanti et al., 2015).

### Cytokines Evaluation

For detection of IL6, IL8, MCP1 and IFNγ the Quantikine ELISA Kit (R&D Systems, RLB00, Minneapolis, MN) was used according to the manufacturer’s instructions (Trubiani et al., 2012a). Supernatants were collected from hDPSCs treated with Curcumin, HEMA 3 and HEMA 5 mmol L^–1^.

### Immunohistochemical Analysis

To study molecules involved in the intracellular signaling, hDPSCs treated with CurLIP, HEMA 3 and HEMA 5 mmol L^–1^ were processed as previously reported by Giacoppo et al. (2017). Anti-human NFkB (1:200, mouse; Molecular Probes), ERK1/2 (1:200, rabbit; Santa Cruz Biotechnology Inc., Santa Cruz, CA, United States) and pERK1/2 (1:50, rabbit; Santa Cruz Biotechnology) were used as primary monoclonal antibodies. Then, samples were incubated for 1 h at 37°C with Alexa Fluor 568 red fluorescence conjugated (1:200, goat anti-rabbit; Molecular Probes, Invitrogen, Eugene, OR, United States) or Alexa Fluor 488 green fluorescence conjugated (1:200, goat anti-mouse; Molecular Probes), as secondary antibody. To stain cytoskeleton actin and nuclei, cells were treated with Alexa Fluor 488 phalloidin green fluorescence conjugate (1:200, Molecular Probes) and TOPRO (1:200, Molecular Probes; Pizzicannella et al., 2011), respectively. Zeiss LSM800META confocal system (Zeiss, Jena, Germany) was used to analyze stained cells, using a Plan Neofluar oil immersion objective (63×). Micrographs were obtained using excitation lines at 488 nm for argon laser beam and at 543 and 665 nm for a helium-neon source.

### Western Blot Analysis

Western blot analysis was performed as previously described by Diomede et al. (2018c). NFkB (1:1000, Molecular Probes), ERK1/2 (Santa Cruz Biotechnology; 1:1000) and pERK1/2 (Santa Cruz 1:750) were used as primary antibodies. β-Actin (Santa Cruz Biotechnology; 1:750) was used to assess the uniform protein loading. Bands analysis has been performed by the ECL method using Alliance 2.7 (UVItec Limited, Cambridge, United Kingdom; Ballerini et al., 2017). Protein bands were quantified with a computer program (ImageJ software).

### RNA Extraction and Quantitative Real-Time PCR (qRT-PCR)

Total RNA was extracted from about 10^6^ cells from each condition by using the NucleoSpin RNA Kit (Macherey-Nagel, Düren, Germany; Diomede et al., 2018a). RNA quantity and quality were assessed by Qubit 2.0 (ThermoFisher Scientific, Whaltam, MA, United States; Diomede et al., 2018b).

1 μg of total purified RNA from each sample was reverse transcribed using the High Capacity RNA-to-cDNA Kit (ThermoFisher Scientific). qRT-PCR was performed in a total volume of 20 μL containing 2× Maxima SYBR Green/ROX qPCR Master Mix (ThermoFisher Scientific), 3 μL of cDNA and 0.3 μM of each primer. GAPDH and HPRT1 were used as housekeeping genes (ThermoFisher Scientific, Waltham, MA, United States; Gugliandolo et al., 2018). Real time amplification conditions were 10 min at 95°C followed by 40 cycles of 15 s at 95°C and 1 min at 60°C. A final melting dissociation curve was run to assess primers specificity. Each sample was run in triplicate. Specific primers pairs (IDT, Skokie, IL, United States) employed are reported in Table 1 The ΔΔCt method and the two-tailed *t*-test were employed to assess the relative gene expression, considering data significant when *p* < 0.05.

**TABLE 1:** Specific primers pairs (IDT, Skokie, IL, United States) employed in the experiments.

**Gene**	**Forward**	**Reverse**
RUNX2	CATCACTGTCCT TTGGGAGTAG	GCCTGGTGGTGTCATTAGAT
DSPP	GAATGGAGCAGATGAGGATGAA	CTGGGTGTCCTCTATTCTTTGG
AXIN2	CTGCCACCAAGACCTACATAAG	GATAGCCACACACGACCTTTAG
COL1A1	CAGACTGGCAACCTCAAGAA	GTTGGGATGGAGGGAGTTTAC
GAPDH	TCTCCTCTGACTTCAACAGCGAC	CCCTGTTGCTGTAGCCAAATTC
HPRT1	CCCTCCCACCCTTTGTTTAT	GTGGATACCTGGAGATTGGTTAG

### Statistical Analysis

Data were analyzed using GraphPad Prism 6.0 (GraphPad Software, La Jolla, CA, United States) with one-way ANOVA test, followed by a Bonferroni *post hoc* test for multiple comparisons. A *p*-value <0.05 was considered statistically significant.

## Results

### Curcumin Encapsulation

**FIGURE 1:**
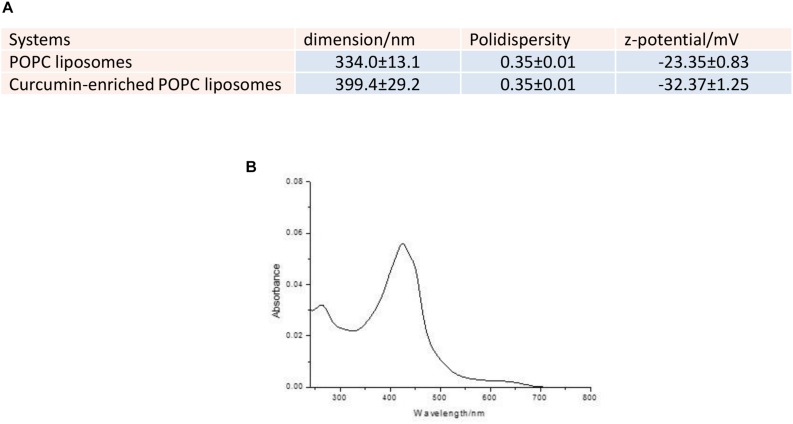
**(A)** Dimensions, polydispersity and zeta potential of the investigated liposomes. **(B)** UV-Vis spectrum of the elution of Sephadex G25 column with milliQ water.

The dimensions of curcumin enriched liposomes were slightly increased from 334 ± 13 to 399 ± 29 nm (Figure 1A). This increase was a clear indication of the fact that curcumin and DMSO did solubilize in the POPC bilayer and was in line with previous measurements (Basnet et al., 2012). The samples demonstrated to be polydispersed as expected from liposomes prepared by sonication protocols. The zeta potential decreased on passing from pure POPC liposomes to curcumin-enriched liposomes, probably due to a partial deprotonation of phenolic moieties (pKa = 8.11 ± 0.46, Calculated using Advanced Chemistry Development (ACD/Labs) Software V11.02) at the used buffered pH and the consequent tendency of dissociated phenoxide groups to expose toward the aqueous bulk. This arrangement, in line with the recently proposed pH-driven, organic solvent-free, liposomal encapsulation of curcumin in liposomes (Cheng et al., 2017), was particularly pronounced in the present experimental conditions as compared to previously investigated curcumin loaded liposomes because we chose to add the curcumin on preformed liposomes. This choice was made because curcumin tends to photodegrade (Chignell et al., 1994) and we used UV irradiation for the sterilization of liposomes before curcumin addition (see Materials and Methods). The entrapment measurements confirmed an entrapment of 95.2% of guest into the bilayer (Figure 1B).

### MTT Assay

**FIGURE 2:**
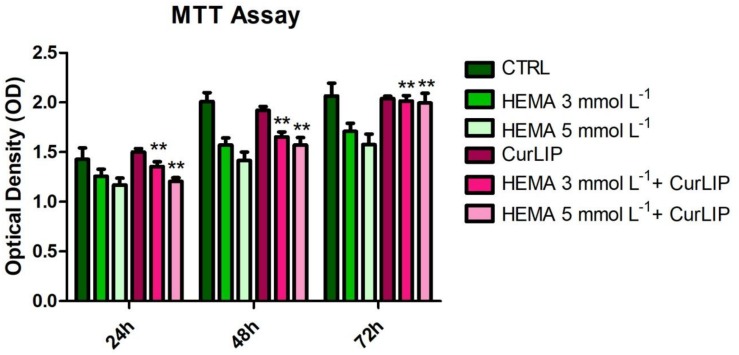
Cell proliferation of hDPSCs treated with CurLIP, HEMA 3 and HEMA 5 mmol L^–1^. Each data point represents the mean of three independent experiments. ^∗∗^*p* < 0.01, hDPSCs co-treated with curcumin and HEMA vs. HEMA treated hDPSCs.

Human DPSCs treated with CurLIP, HEMA 3 and HEMA 5 mmol L^–1^ were evaluated with MTT assay after 24, 48 and 72 h. In Figure 2, the data showed that the HEMA 5 mmol L^–1^ treated sample had the lowest proliferation rate, compared with other samples. On the other hand, cells subjected to the combined treatment with CurLIP/HEMA 3 or 5 mmol L^–1^ showed a proliferation rate similar to the control cells. The CurLIP treatment alone did not change the cell proliferation rate when compared to the control cells.

### Cytokines Release Assessment

**FIGURE 3:**
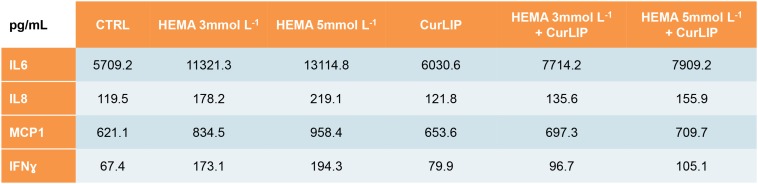
Cytokines differentially released from hDPSCs in the supernatants after treatment with CurLIP, HEMA 3 and HEMA 5 mmol L^–1^.

The analysis of released cytokines in the culture medium of the hDPSCs treated with CurLIP, HEMA 3 and HEMA 5 mmol L^–1^ was performed by RayBiotech. The quantitative method showed an increase of the IL6, IL8, MCP1 and IFNγ in hDPSCs treated with HEMA 3 and HEMA 5 mmol L^–1^, meanwhile the cells co-treated with CurLIP/HEMA 3 or HEMA 5 mmol L^–1^ showed a decreasing level of cytokines similar to that observed in control cells. The data confirmed that HEMA treatment induced an inflammatory stimulus changing the pro-inflammatory cytokines release in cells treated with both concentrations of HEMA (Figure 3).

### Immunofluorescence Analysis

**FIGURE 4:**
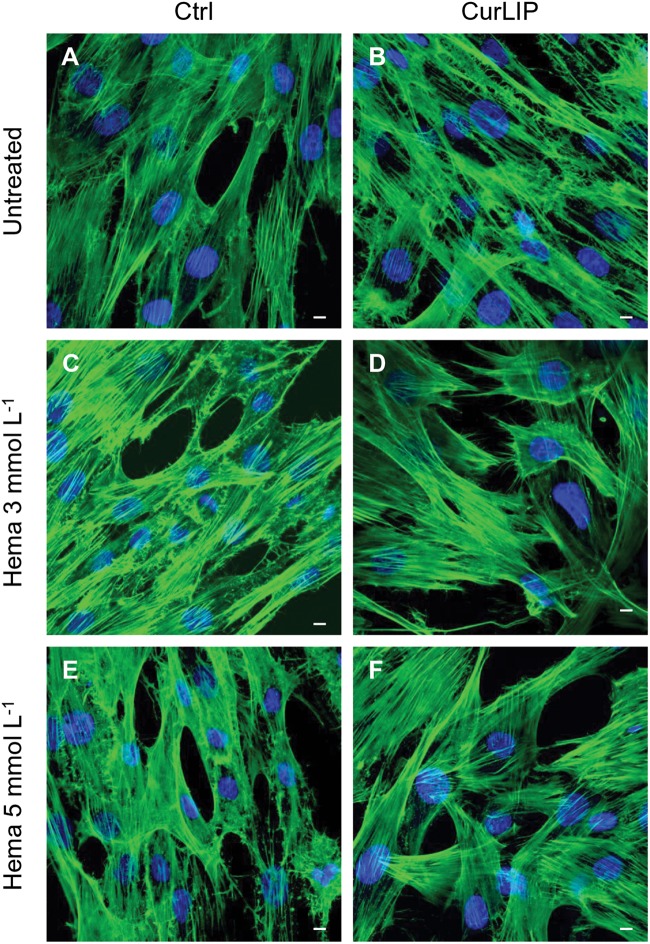
Representative confocal images of phalloidin 488 (green) and TOPRO (blue) fluorescent staining showed cells morphology in different culture conditions. **(A)** Untreated cells; **(B)** CurLIP treated cells; **(C)** HEMA 3 mmol L^–1^ treated cells; **(D)** HEMA 3 mmol L^–1^ with CurLIP treated cells; **(E)** HEMA 5 mmol L^–1^ treated cells; **(F)** HEMA 5 mmol L^–1^ with CurLIP treated cells. Mag: 40×. Scale bars: 10 μm.

Untreated hDPSCs showed, at confocal microscopy observation, a typical fibroblast-like morphology. In CurLIP treated cells, extremely fine filamentous filopodia could be observed as compared to the control cells. The treatment with HEMA 3 and HEMA 5 mmol L^–1^ modified the cellular features as visible in Figure 4. Cells showed small and short cytoplasmic processes. The addition of the CurLIP solution to cells treated with HEMA 3 or HEMA 5 mmol L^–1^ seemed to have a protective effect, in fact cells showed a morphology quite similar to the control cells.

**FIGURE 5:**
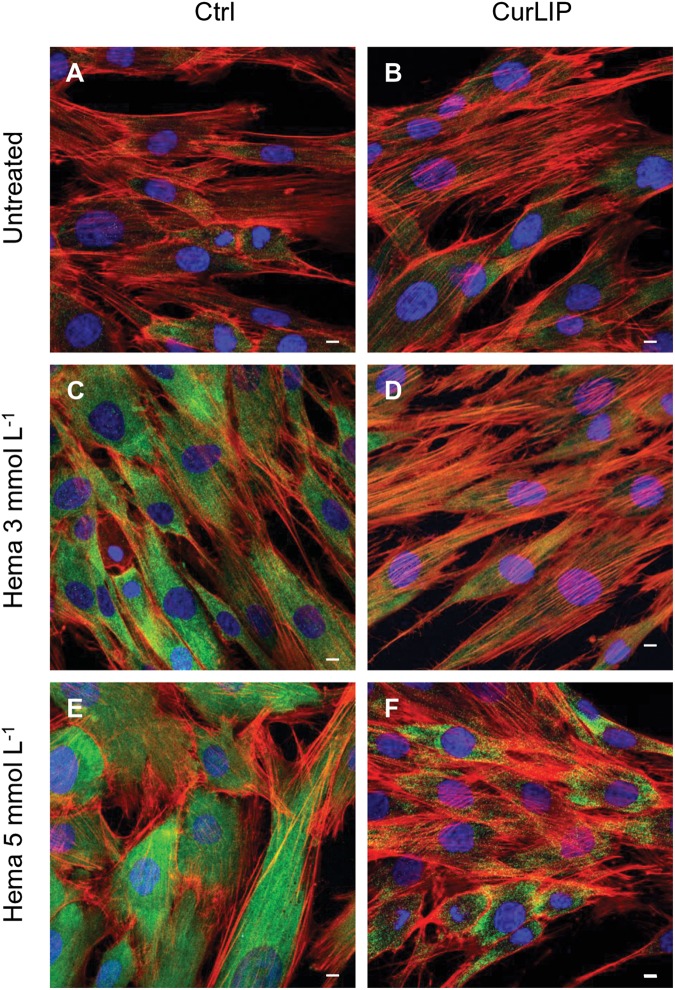
Representative confocal images of NFkB (green), phalloidin 568 (green) and TOPRO (blue) fluorescent staining showed cells morphology in different culture conditions. **(A)** Untreated cells; **(B)** CurLIP treated cells; **(C)** HEMA 3 mmol L^–1^ treated cells; **(D)** HEMA 3 mmol L^–1^ with CurLIP treated cells; **(E)** HEMA 5 mmol L^–1^ treated cells; **(F)** HEMA 5 mmol L^–1^ with CurLIP treated cells. Mag: 40×. Scale bars: 10 μm.

**FIGURE 6:**
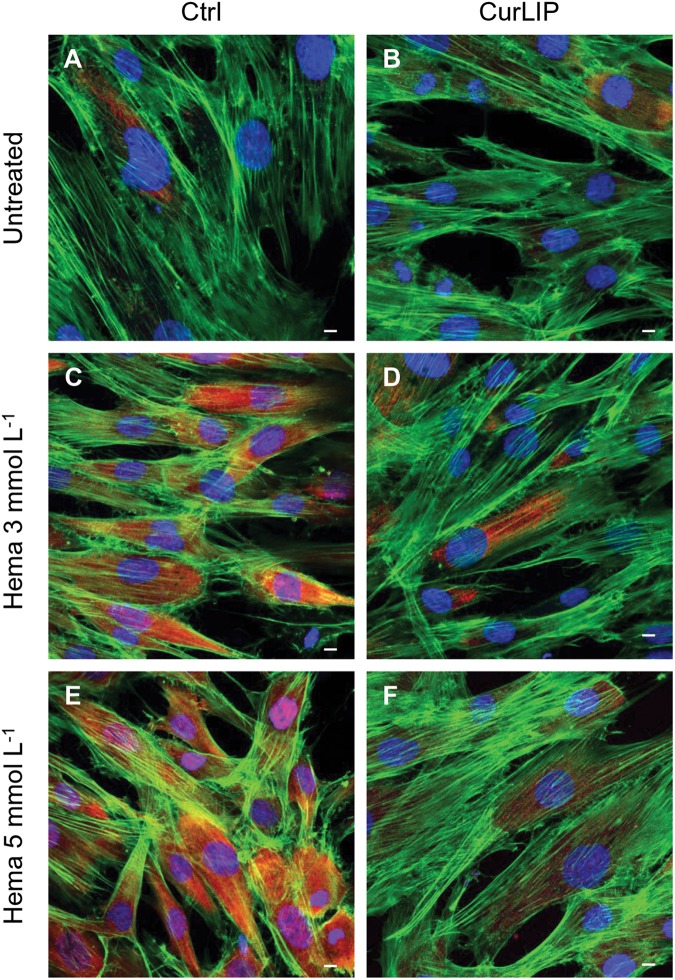
Representative confocal images of ERK (red), phalloidin 488 (green) and TOPRO (blue) fluorescent staining showed cells morphology in different culture conditions. **(A)** Untreated cells; **(B)** CurLIP treated cells; **(C)** HEMA 3 mmol L^–1^ treated cells; **(D)** HEMA 3 mmol L^–1^ with CurLIP treated cells; **(E)** HEMA 5 mmol L^–1^ treated cells; **(F)** HEMA 5 mmol L^–1^ with CurLIP treated cells. Mag: 40×. Scale bars: 10 μm.

**FIGURE 7:**
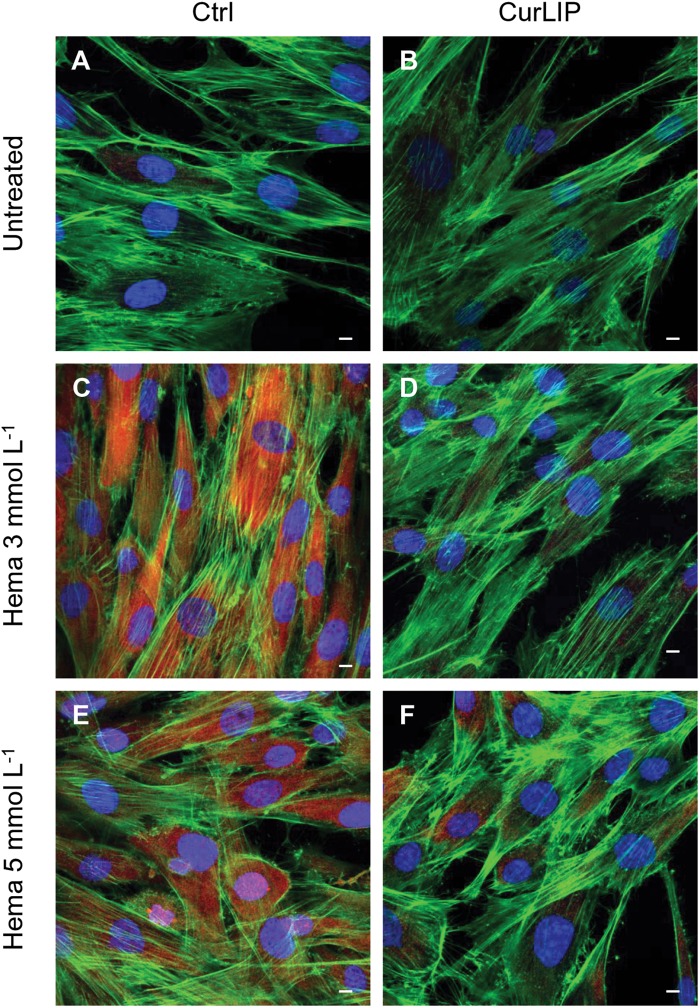
Representative confocal images of pERK (red), phalloidin 488 (green) and TOPRO (blue) fluorescent stains showing the cells morphology in different culture conditions. **(A)** Untreated cells; **(B)** CurLIP treated cells; **(C)** HEMA 3 mmol L^–1^ treated cells; **(D)** HEMA 3 mmol L^–1^ with CurLIP treated cells; **(E)** HEMA 5 mmol L^–1^ treated cells; **(F)** HEMA 5 mmol L^–1^ with CurLIP treated cells. Mag: 40×. Scale bars: 10 μm.

Immunofluorescence staining demonstrated the expression of NFkB, ERK and pERK. The treatment with HEMA 3 and HEMA 5 mmol L^–1^ showed a high expression of NFkB, ERK and pERK while the cells cultured with CurLIP before HEMA 3 and HEMA 5 mmol L^–1^ exposure showed a restoration to the conditions of the untreated cells (Figures 5–7). In particular HEMA 3 and HEMA 5 mmol L^–1^ treatment induced a nuclear translocation of NFkB (Figures 5C,E). CurLIP treatment restored the expression of NFkB at cytoplasmic level (Figures 5D,F).

### Western Blot Analysis

**FIGURE 8:**
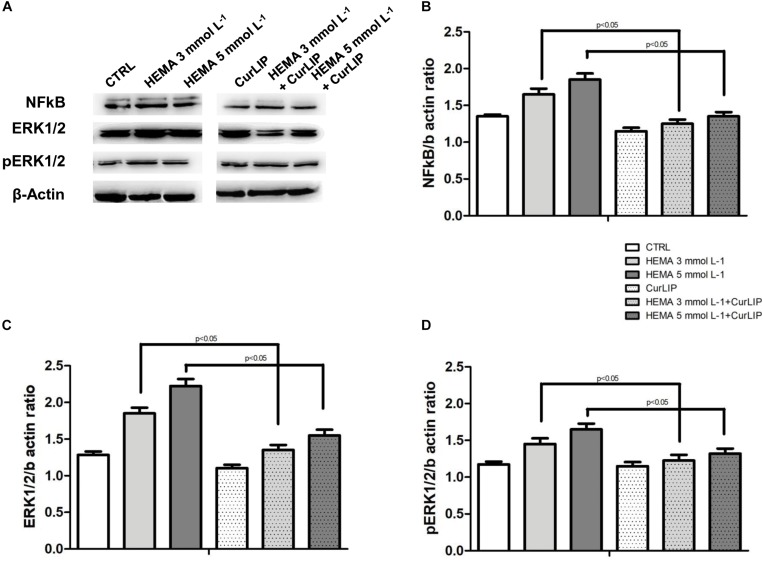
**(A)** The western blotting analysis with anti-NFkB, ERK and pERK in treated and untreated cells. β-actin as internal control is shown. Densitometric analyses of western blot specific bands, **(B)** NFkB, **(C)** ERK and **(D)** pERK, respectively.

The variations in NFkB, ERK and pERK protein expression were further evaluated by the western blot analysis after treatment. As shown in Figure 8, the samples exposed to HEMA 3 or HEMA 5 mmol L^–1^, showed an up regulation of NFkB, ERK and pERK whereas cells co-treated with CurLIP showed a down regulation of the same proteins. These results suggested that CurLIP treatment reduce the HEMA effects on hDPSCs (Figure 8).

### Gene Expression

**FIGURE 9:**
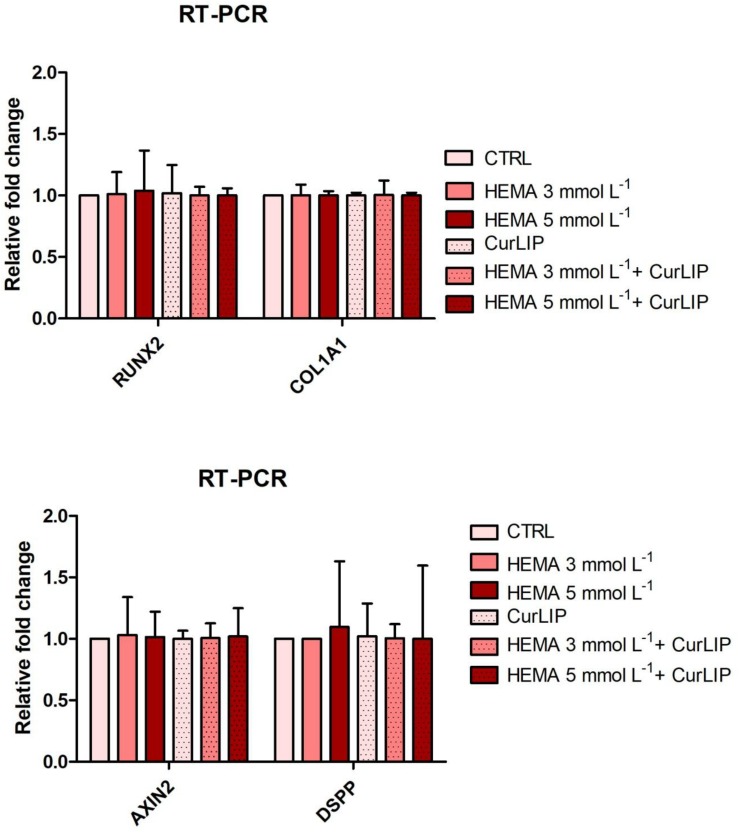
qRT-PCR analysis of RUNX2, COL1A1, DSPP, and Axin2.

qRT-PCR analysis showed no significant differences among the analyzed conditions for all tested genes. Results were reported in Figure 9 and showed no differences in the expression of Runt-related transcription factor-2 (RUNX2), Collagen1A1 (COL1A1), Dentin SialoPhosphoProtein (DSPP) and Axin related protein-2 (AXIN2).

## Discussion

Progress in dentistry is associated with the innovation of dental materials and the use of new molecules for regenerative therapies, inter alia the design of new materials for restorative dentistry or for endodontic therapy.

Resin-based methacrylates, common materials widely used in restorative dentistry, are viscous substances that are converted into solid material trough polymerization processes (Trubiani et al., 2012c). The polymerization is, often, incomplete, and induces the release of monomers into oral cavity, in dentin micro-channels and finally the bloodstream, inducing an inflammatory process. This inflammatory process may alter the odontoblast activity and is associated to pulp tissue homeostasis damage (Trubiani et al., 2012b). Aim of regenerative endodontics is to rebuild the original pulp tissue based on tissue engineering principles (Murray et al., 2007; Diogenes and Hargreaves, 2017). Recently, biomimetic scaffolds, loaded with natural molecules, have been generated to stimulate pulp tissue regeneration and to prevent inflammatory processes (Fioretti et al., 2011).

Extensive research in the past half century demonstrated that curcumin, a food component, used as a spice and food coloring agent, is also used as antioxidant, anti-inflammatory, and anticancer compound that exerts antimicrobial effects (Shishodia et al., 2007). In particular, the bactericidal activity showed a synergistic effect with antibiotics for several pathogens (Packiavathy et al., 2013; Roudashti et al., 2017). Studies carried out in other laboratories have identified a number of different molecules involved in inflammation that are inhibited by curcumin such as phospholipase, lipoxygenase, cyclooxygenase 2, leukotrienes, thromboxane, prostaglandins, nitric oxide, collagenase, elastase, hyaluronidase, monocyte chemoattractant protein-1 (MCP1), interferon-inducible protein, tumor necrosis factor (TNF), and interleukin (IL) 12 (Chainani-Wu, 2003). Curcumin is pharmacologically safe. In fact, clinical trials showed that the curcumin can be administered to the patients up to 8,000 mg/day without adverse effects. The pharmacokinetics of curcumin-loaded liposomal gel showed that nano-sized liposomes are able to penetrate within 1 h in both stratum of corneum and skin (Madan et al., 2018).

Starting from these concepts the aim of the present study was to evaluate the ability of curcumin, used at a concentration of 20 μM (Xiao et al., 2018), to restore the homeostasis of dental pulp cells in response to HEMA treatment. HEMA is reported to induce DNA damage, apoptosis and necrosis in various cell lines *in vitro*, but generally concentrations above 4 mmol L^–1^ are necessary to induce these effects (Ansteinsson et al., 2011). In our experiments we have tested the inflammatory response to HEMA 3 and 5 mmol L^–1^.

There is an increasing interest in the tissue’s regeneration for the use of autologous stem cells isolated from various adult tissues due to the fewer ethical concerns as compared to embryonic stem cells (Spina et al., 2016). Oral cavity is an easily accessible source of mesenchymal stem cells, as periodontal ligament, gingival and dental pulp (Stellavato et al., 2017). Mesenchymal stem cells are able to maintain the stem cells phenotype and the capacity to differentiate *in vitro* toward osteogenic, adipogenic and osteogenic commitment (Libro et al., 2016; Manescu et al., 2016). In particular, human dental pulp stem cells, represent a relevant cellular model constituted of a cell population homologous to the primary tissue with a large capacity of proliferation, the ability to maintain their cellular phenotype for a long time period, until 15 passage (Diomede et al., 2017a), and the possibility to undergo toward mesenchymal phenotype (Pizzicannella et al., 2018a).

The cell proliferation of hDPSCs treated with HEMA at different concentrations, associated to cell morphology observation, was measured by MTT assay. Reduction of cell proliferation and alteration of cell morphology have been directly associated with the HEMA treatment (Trubiani et al., 2010). CurLIP treatment was able to restore cell proliferation and morphological features: hDPSCs co-treated with curcumin and HEMA displayed conditions more similar to control cells than hDPSCs treated only with HEMA.

HEMA 3 and 5 mmol L^–1^ stimulated an upregulation of IL6, IL8, MCP1, and IFNγ inflammatory molecules secretion, while CurLIP treatment significantly inhibited the expression of the above mentioned inflammatory cytokines.

IL6 is a pleiotropic cytokine playing an active role in immune responses and in the development of the acute phase response (Heinrich et al., 1990). *In vivo*, the increased secretion of IL6 promotes monocyte differentiation into mobile and active macrophages able to secrete MCP1 and Metalloproteinases (MMPs), facilitating the invasion from the adventitia into the media (Kagan and Medzhitov, 2006) and it is also involved in root repair (Oliveira et al., 2018).

IFNγ synchronizes a diverse array of cellular programs through transcriptional regulation of immunologically relevant genes (Schroder et al., 2004).

IL8 is a chemokine that promotes the recruitment and activation of neutrophils to the sites of acute inflammation, where neutrophils not only kill bacteria by phagocytosis, but also destroy affected tissue by secreting proteases and generating oxidative oxygen species (Suzuki et al., 2008).

IL6 and IL8 cytokines secretion is related to increased activation of the transcription factor NFkB. Previously we have demonstrated that LPS-Gingivalis (G) inflammatory stimulus provokes, in endothelial cells, the release of IL6, IL8, and MCP1 molecules and the activation of signaling network Toll Like Receptor 4 (TLR4)/NFkB/ERK/pERK (Diomede et al., 2017b; Pizzicannella et al., 2018b).

HEMA treatment triggers the above-mentioned cytokines, highlighting the expression of an inflammatory pathway, induced by Lipopolysaccharide from LPS-G and our results give evidence, for the first time, that CurLIP treatment reduces the NFkB activation pathway inducing a down regulation of ERK and pERK signaling.

Taking these experiments in consideration and with the limitation of this study, we retain that CurLIP formulation reduces the inflammatory level in HEMA treated dental pulp, indicating for the complex CurLIP a new role as a therapeutic potential in dental pulp regeneration.

Following these evidences, we have analyzed, at molecular level, the involvement of CurLIP in odontoblast differentiation. At this purpose, the expression of odontoblast-related marker genes such as RUNX2, COL1A1, Axin2, and DSPP have been characterized. RUNX2 and COL1A1 possess a decisive role during mineralization process in the early step of mineralized tissue formation (Diomede et al., 2016b). DSPP is a positive regulators of hard tissue mineralization (Suzuki et al., 2012), acting as nucleators of apatite crystal formation in the presence of collagen. DSPP is able to induce highly organized intrafibrillar collagen mineralization other than play an important role during odontoblasts differentiation (Deshpande et al., 2011). Axin2 is upregulated in response to tooth damage and induces the secretion of reparative dentine (Babb et al., 2017). Despite tooth damage or trauma lead new odontoblast-like cells recruitment, little is known about the molecular events involved in the response to dentine damage. RT-PCR provided an evidence of no increase of the above-mentioned genes. This data offers the opportunity to speculate that CurLIP is able to provide a “niche” environment for cells replacement, as demonstrated through the increase of cell proliferation. Then, findings indicate that CurLIP treatment is able to reduce inflammatory process via NFkB/ERK/pERK signaling, but it is not involved in the odontoblastic phenotype differentiation. At the same time HEMA treatment may interfere with the regulation of intracellular homoeostasis, leading to the reduction in cell viability in agreement with previous evidences that demonstrated the capacity of HEMA to inhibit the migration and differentiation of pulp cells into the odontoblast layer *in vitro* (Williams et al., 2013; Pizzicannella et al., 2019).

The present conclusion suggests that CurLIP formulation provides a new approach for functional tissue/organ restoration and may represent a promising therapeutic strategy in the design of innovative endodontic procedure, significantly improving the quality of dental care with a major human community impact. CurLIP treatment could be considered a promising alternative in regenerative endodontics practice.

## Ethics Statement

Ethical Committee at the Medical School, “G. d’Annunzio” University, Chieti, Italy (number 266/2014) has approved the present study. All patients have signed the informed consent as requested by rules of the Department of Medical, Oral and Biotechnological Sciences (ISO 9001:2008, RINA certified 32031/15/S).

## Author Contributions

BS, JP, and FD designed the study, performed the experiments, analyzed the data, and wrote the manuscript. RZ and AF developed the liposome formulation and revised the manuscript. MDA and VG performed RT-PCR analysis and revised the manuscript. OT designed the study, analyzed the data, and wrote and revised the manuscript.

## Conflict of Interest Statement

The authors declare that the research was conducted in the absence of any commercial or financial relationships that could be construed as a potential conflict of interest.
